# The Relationship among Physical Activity, Intestinal Flora, and Cardiovascular Disease

**DOI:** 10.1155/2021/3364418

**Published:** 2021-10-12

**Authors:** Qiuyu Yan, Wenhui Zhai, Chenghao Yang, Zihao Li, Longfei Mao, Mingyi Zhao, Xiushan Wu

**Affiliations:** ^1^Guangdong Cardiovascular Institute, Guangdong Provincial People's Hospital, Guangdong Academy of Medical Sciences, Guangzhou, Guangdong 510100, China; ^2^Department of Pediatric, The Third Xiangya Hospital, Central South University, Changsha 410013, China; ^3^Xiangya School of Medicine, Central South University, Changsha, 410013 Hunan, China; ^4^Bioinformatics Center, College of Biology, Hunan University, Changsha, 410082 Hunan, China; ^5^The Center for Heart Development, State Key Laboratory of Development Biology of Freshwater Fish, Key Laboratory of MOE for Development Biology and Protein Chemistry, College of Life Sciences, Hunan Normal University, Changsha, 410012 Hunan, China

## Abstract

Cardiovascular diseases (CVDs), which are associated with high morbidity and mortality worldwide, include atherosclerosis (AS), hypertension, heart failure (HF), atrial fibrillation, and myocardial fibrosis. CVDs are influenced by the diversity, distribution, and metabolites of intestinal microflora, and their risk can be reduced through physical activity (PA) such as regular exercise. PA benefits the metabolic changes that occur in the gut microbiota (GM). The major metabolites of the GM influence pathogenesis of CVDs through various pathways. However, the relationship between PA and GM is less well understood. In this review, we discuss the impacts of different types of PA on intestinal microflora including the diversity, distribution, metabolites, and intestinal barrier function including intestinal permeability, with a focus on the mechanisms by which PA affects GM. We also discuss how GM influences CVDs. Finally, we summarize current research and knowledge on the effects of PA on CVD via regulation of the GM and intestinal function. More understanding of relevant relationship between PA and GM may provide hope for the prevention or treatment of CVDs. Furthermore, a better understanding of regulation of the GM and intestinal function may lead to novel diagnostic and therapeutic strategies, improving the clinical care of CVD patients.

## 1. Introduction

CVDs are a group of circulatory system diseases or disorders that include AS, hypertension, HF, atrial fibrillation, and myocardial fibrosis. According to numerous statistics, CVDs account for one-third of all deaths worldwide [1]. A recent study discovered that a majority of CVDs is attributable to dietary risks, high systolic blood pressure (SBP), high body mass index(BMI), high total cholesterol (TC) level, high fasting plasma glucose (FPG) level, tobacco smoking, and low levels of PA [1]. All of these factors promote the occurrence and progression of CVDs.

The term “gut microbiota” refers to the hundreds of billions of bacterial microorganisms found in the human digestive tract, including bacteria, fungi, viruses, archaea, and protists [2]. Among them, the intestinal bacteria are the most well-studied, whereas the etiologic role of the virome and archae in human health remains largely unexplored. The bacteria contain around 9 million genes [3], making it 150 times larger than the human genome [4].

The normal flora performs numerous critical physiological functions, such as maintaining the balance between human body and microorganism, promoting the function of immune system and clearance of cell debris, draining toxins out of the body, and providing nutrition [5]. There has been a recent surge of interest in understanding the role of human intestinal microflora in CVDs. The metagenome-based association study has yielded a plethora of information about the presence of CVD-associated GM [6]. The diversity, distribution, and metabolites of intestinal microflora have all been reported to influence CVD. There were also some links found between intestinal microflora, intestinal barrier function, intestinal inflammation, and CVD.

Meanwhile, lifestyle modifications such as diet and exercise have an impact on the composition of GM as well as host's health. Compared with diet research, there is still a lack of research on the relationship between PA and GM. Regular PA may reduce the incidence of CVD and offer a beneficial effect on health by disrupting systemic homeostasis [7]. For example, some studies have shown that PA can reduce CVD risk by promoting healthy phenotypic distribution of intestinal microflora. Although PA has been commended by many guidelines and expert consensus for its prevention and protection effects on CVD (especially arteriosclerotic cardiovascular disease), many details of its mechanisms remain unknown. Subsequently, studying the relevant relationship between PA and GM should be beneficial in the treatment of CVDs.

This review describes the effects of different types of exercise on intestinal microflora and how PA affects GM. The impact of intestinal microflora on different CVDs such as hypertension, AS, and HF is discussed. Finally, we analyze the current knowledge on the effects of PA on CVD through modulation of the intestinal microflora and intestinal function and how we may apply these to future research and medical treatment.

## 2. Effects of PA on GM

Exercise can effectively prevent some chronic diseases related to gut microbiota disorders, such as obesity, hypertension, diabetes, respiratory diseases, and cardiovascular diseases [8]. Therefore, it has long been speculated that exercise is linked to an altered GM. Recent animal studies have shown that exercise training can modify the gut of rodents and their associated gut microbiome [9] [10]. A study also found that exercise training can alter GM of those with an obese and hypertensive phenotype. Other animal studies have indicated that exercise-induced changes in the gut microbiome may be involved in the modulation of high fat-diet, experiment diabetes, and biphenyls-induced dysbiosis [11] [12] [13]. In a study comparing the GM of male rugby players with healthy controls, it was found that the number and diversity of GM of professional rugby players were significantly higher than that of the general population with the same age and BMI [14]. This could be due to the fact that increasing protein intake through exercise training can promote the diversity of GM [15].

### 2.1. Influence on Intestinal Microecological Environment

Intestinal microecology is composed of GM and the gut environment they dwell and has played an important part in the normal operation of the system. Many findings have shown that physical activity induces metabolic changes in the intestinal tract and increases intestinal permeability.

#### 2.1.1. Metabolome

Based on correlations between endurance running and GM modifications found in a study [16], metabolic changes in the gut environment can be caused by endurance exercise, rapidly and strikingly. The authors found a total of 40 fecal metabolites significantly altered considerably after finishing a half-marathon race. The most significantly changed metabolites were organic acids (the major increased metabolites) and nucleic acid components (the major decreased metabolites). Similar to Sedoheptulose 7-phosphate and L-Homotyrosine, aminomalonic acid, was the key environmental variable influencing the variation of GM after running.

Previous studies in rat by Huang et al. found that the rate of tricarboxylic acid cycle and antioxidant activity elevated following endurance training [17]. In addition, some hormonal markers, such as insulin, leptin, and adiponectin, showed unique patterns after a marathon running [18] [19]. Moreover, fecal concentrations of short chain fatty acids (SCFAs) were aggrandized in lean, but not obese people after running. Changes in SCFAs were observed concomitant to an exercise-induced increase in the relative abundance of the butyrate-regulating gene BCoAT and the propionate­regulating gene mmDA, while propionate and butyrate declined towards baseline levels. In the obese group, concentrations of all three SCFAs did not change from E6 to W6. Furthermore, metabolic products are firmly associated with GM alterations. For example, the increase in the bacterial taxa *Coriobacteriaceae* after running has been shown to correlate with 15 differential metabolites: 4 nucleic acid components, 4 organic acids, 2 steroids, 1 amino acid, and 4 other metabolites [20].

Thus, it may be argued that exercise can change metabolome in the gut environment via some processes that have yet to be elucidated.

#### 2.1.2. Influence on Intestinal Permeability

Besides changes of metabolome, many studies have demonstrated that intestinal permeability can be enhanced by exercise through a variety of mechanisms. First, physical trauma in intestinal epithelial cell is associated with an increase in intestinal permeability. After physical shock, such an endurance running, the intestinal tract is continuously subjected to mechanical impact from the moving body, resulting in the disruption of intestinal epithelial integrity and an increase in intestinal permeability [14].

Second, increased intestinal permeability is associated with changes in the composition and metabolism of the intestinal microbiota after PA. For instance, secondary bile acids are thought to have signaling functions including promotion of gut barrier integrity [21]. Similarly, *Actinobacteria* phyla species including those from the *Bifidobacterium* and *Collinsella* genera show anti-inflammatory and immunomodulatory properties that may preserve the intestinal barrier and influence the intestinal permeability during the PA [22]. *Proteobacteria*, on the other hand, have been related to inflammation, which may increase the intestinal permeability [23]. These findings show that alterations in the composition and metabolism of the intestinal microbiota can affect intestinal permeability during physiologic stress by altering the availability of amino acid precursors and secondary bile acids.

Third, the rise in intestinal permeability is associated with an increased inflammation. Intense or prolonged exercise can impair splanchnic perfusion and cause stress-induced muscle damage which trigger intestinal hypoxia, inflammation, and oxidative stress that collectively degrade intestinal barrier integrity and increase intestinal permeability [24].

Last, intestinal permeability is influenced by variety of other factors. For example, prolonged intestinal hypoperfusion causes enterocyte injury [25]. Also, increased intestinal permeability is likely to be caused by the phosphorylation of several tight junction proteins [26].

In short, we can conclude that intestinal permeability can be increased by physical activity through several mechanisms such as inflammation.

### 2.2. Improvement of GM by PA

In spite of influence on intestinal microecological environment, the state of gastrointestinal microorganisms can be improved by physical activity. As is known to all, the gastrointestinal microorganisms are intimately linked to nutrient uptake, vitamin synthesis, energy harvesting, inflammatory modulation, and host immune response, which collectively contribute to human health. Many factors, including age, diet, and exercise, influence the GM. Some authors have provided compelling evidence that exercise can have a significant impact on gut microbial communities assignably. Many animal studies have demonstrated that exercise can prevent unhealthy states by altering the GM. Lambert et al. found that *Firmicutes* were more abundant and the rate of *Bacteroides/Prevotella* genera was lower in mice after exercise [27]. Evans et al., on the other hand, claimed that exercise increased the *Bacteroidetes* but decreased *Firmicutes* in mice [28]. June et al. also reported that an increase in phylum *Firmicutes* but decrease in phyla *Tenericutes* and *Bacteroidetes* was observed in mice after wheel running [29]. A study of professional athletes concluded that exercise may increase the *α*-diversity of GM and abundance of the bacterial genus *Akkermansia* [14].

However, a simple abstract of how exercise affects the GM cannot convey the complex relationship between exercise and the GM. The types of exercise and the conditions in which it is performed have a significant impact on the results. The following sections discuss the impacts of voluntary exercise, forced exercise, endurance exercise, and high-intensive exercise on the GM, as well as the potentially relevant mechanisms.

#### 2.2.1. Effects of Voluntary Exercise on GM

The study of mice's changes following voluntary wheel running (VWR) or forced treadmill running (FTR) revealed that voluntary exercise reduced the richness of the microbial community but increased the distribution of bacterial communities [30]. This result generated a discrepancy between the widespread perception that decreased bacterial population richness was associated with disease states in the majority of cases and the conclusion that the voluntary exercise can always benefit people's health. However, this is similar to several antidiabetic medicines, implying that voluntary exercise can have an impact on diabetes. In addition, several *Firmicutes* bacteria are selectively increased, and the percentage of *Bacteroidetes/Prevotella* changed, which is related to obesity relief [27]. Voluntary exercise has a considerable impact on the relative balance of the two major bacterial phyla, *Bacteroidetes* and *Firmicutes*, as well as the prevention of diet-induced obesity and normalization in glucose tolerance [31]. What is more, some studies demonstrate that following voluntary exercise, the concentration of n-butyric acid salt rises significantly more than mice staying sedentary, implying that the number of bacteria generating n-butyric acid salt increased, which has anti-inflammatory properties. The *Turicibacter* is an important genus to mention because it is significantly reduced following voluntary exercise. Meanwhile, Kellermayer et al. claimed that the particular genus was associated with immune system [32], and some studies have showed that it played a role in the pathogenesis of inflammatory bowel disease. Thus, we can make the bold claim that this bacteria population will be of crucial importance in future research on exercise and gastrointestinal health.

#### 2.2.2. Effects of Forced Exercise on GM

At the moment, few studies have been conducted to determine how forced exercise influences the GM and gastrointestinal health. Due to the fact that forced exercise is less prevalent in daily life than voluntary exercise, so the role of forced exercise is less well defined. In contrast to voluntary exercise, analyzed using PCoA, the FTR groups cluster distinctly at both intestinal sites (i.e., the distal colon and the cecal contents), as determined by the unweighted UniFrac distance metric. As previously speculated from the study above, the community richness increased, while evenness decreased in the FTR group compared with secondary home cage control (SED) group [29]. Additionally, it was found that *Tenericutes* and *Proteobacteria* were elevated in the feces of the FTR groups, which can provide LPS to gut epithelial cells and immunological cells, resulting in the development of some diseases [29]. In the Matsumoto study, forced running increased cecal n-butyrate concentration because of a matching change in the n-butyrate-producing bacteria, as compared to sedentary rats. Additional trials are required to be done to advance our understanding of the interactions between forced exercise and GM.

#### 2.2.3. Effects of Endurance Exercise on GM

Numerous studies have been conducted over last decades to determine how endurance exercise affects the GM. It was found that endurance exercise may cause metabolic alterations in blood, urine, muscles, and lymph that potentially have an effect on GM within several hours. Zhao et al. [16]. suggested that long-distance endurance running can rapidly induce metabolic changes in the gut environment, to which the GM respond by adjusting certain bacteria, as shown by a study based on an untargeted metabolomics methodology and 16S rDNA sequencing analysis. It can evident that endurance running has little effect on GM's *α*-diversity, although the abundances of several bacterial taxa can be significantly different after endurance exercise. After running, the authors found 26 operational taxonomic units (OTUs) in the AFT (after running) group and 15 special operational taxonomic units in the BEF (before running) group. Also, we observed a considerable increase in species richness following running in the *Coriobacteriaceae* and *Succinivibrionaceae* families. Besides, the “cell motility” function of GM was induced after running, but “energy production and conversion” was repressed. Taniguchi et al. [33] similarly investigated how endurance exercise affected the GM in elderly adults. After five-week endurance exercise, the change in the diversity of GM was comparable to interindividual variation. It is noteworthy that changes in alpha diversity were negatively correlated with changes in systolic and diastolic blood pressure, and the relative abundance of *Clostridioides* difficile decreased, whereas that of *Oscillospira* increased, all of which all are associated with cardiometabolic factors. Allen et al. [34] conducted an experiment in which lean and obese subjects underwent six weeks of endurance training with diet controlled followed by a six-week washout period, and they found that exercise-induced alterations in the microbiota were completely reversed once exercise training ceased after the washout period. In the future, we believe that more explorations will scramble to place in the future.

#### 2.2.4. Effects of High-Intensive Exercise on GM

To identify how exercise of different intensities with similar energy consumption affect the GM, Kern et al. [35] conducted over an intervention in which obese patients were divided into four groups—habitual living, active commuting by nonmotorized bicycle, leisure-time exercise of moderate intensity, and vigorous intensity exercise. This study characterized that beta diversity was reduced in the high intensity group compared with control. By contrast, the high exercise group experienced a greater increase in alpha diversity at three months. What is more, the Bacteroidetes grew in number, but the *Firmicutes*/*Bacteroidetes* ratio decreased. Recent research by Arakawa et al. [18] suggested that while short-term high intensity interval training had no effect on the overall composition and diversity of the GM, certain microbiome genera, associated with insulin sensitivity markers which were improved by high intensity interval training in overweight participants, were more abundant. All these taxa like *Coprococcus_3*, *Blautia*, *Lachnospiraceae_ge*, and *Dorea* derived from the *Firmicutes* phylum and *Clostridiales* order.

When comparing the two studies above, that exercise-induced alterations on the GM are dependent on obesity status and that weight loss during exercise is a confounding interference factor which can affect the overall effects.

In summary, different types of PA have relatively different influence on the GM with one or more of the same or different mechanisms.

### 2.3. Potential Mechanisms of How PA Influence the GM

Generally speaking, the mechanisms by which PA alters diversity, structure, abundance, and functions of the GM are not entirely obvious and definite at the moment. Many factors, both internal and external, contribute to the formation of GM collectively. We shall discuss various possible mechanisms in this section.

Exercise-induced metabolites can provide energy and nutrition to certain bacteria taxa or may have some physiological effects, hence influencing the diversity and abundance of the GM. For example, numerous studies have indicated an association between stool metabolics and the stool microbiota. Also, there is a relationship between exercise-induced improvements in insulin sensitivity and certain bacterial taxa in overweight men. The abundance of the bacterial taxa *Coprococcus_3*, *Blautia*, *Lachnospiraceae_ge*, and *Dorea* is improved after high intensity interval training [18]. Moreover, previous studies have demonstrated that the *Coriobacteriaceae* family is involved in bile acid metabolism and that the proportion of secondary bile acids in feces is reduced after voluntary wheel running [14].

Another vital aspect affecting the GM is the pH in the gut environment, which can result in alterations in the structure and composition of the GM. IT has been found that GI transit time has an effect on the pH within the colonic lumen. In addition, repeated aerobic exercise has been shown to improve GI transit time in healthy individuals and middle-aged patients with chronic constipation [36] [37]. Furthermore, aerobic exercise may increase fecal SCFA concentration which results in a reduction in pH in the colonic lumen [38].

PA can stimulate the hypothalamic-pituitary-adrenal axis, which results in the production of various hormone such as noradrenaline and glucocorticoid, disrupting GM's previous equilibrium. It is illustrated that corticotropin-releasing factor could change gastric acid, gastric motility, and digestive juice [39]. Moreover, the increase of noradrenaline leads to significant decrease in lactic acid bacteria and increase in virulence of pathogenic enterobacteria in the intestinal tract [40].

To sum up, each of these functions may play a part in the GM alterations. Additional and severe interventions are thus required, and the idea that changes in the GM result from integrated element, just one or a few, is extremely significant for us to consider.

## 3. Effect of GM on CVD

### 3.1. GM and Hypertension

Hypertension has long been a research hotspot due to its role as a key risk factor for stroke and coronary heart disease morbidity and mortality, as well as one of the most common CVDs. Long-term high blood pressure can affect the function of organs such as the heart, brain, and kidney, eventually resulting in their failure. Recent studies have shown that there is a close relationship between GM and hypertension. It has been pointed out in the spontaneously hypertensive rat model, it was observed that the richness, diversity, and evenness of intestinal flora were significantly decrease, and the proportion of *Firmicutes/Bacteroidetes* ratio increased significantly. These changes were accompanied by decreases in acetate-and butyrate-producing bacteria [41]. There also have been studies of feeding a high-salt diet-induced hypertensive rat model, using 16S rRNA gene mutation technology to study the cecum samples of Dahl salt-sensitive (S) and Dahl salt-resistant (R) rats, comparing the gut microbial colon the differences in structural characteristics indicate that the family *S24-7* of the phylum *Bacteroides* and the family *veillonellaceae* in the phylum *Bacteroides* are higher in the S rats compared with the R rats [42].This has indicated a link between GM and hypertension. In a study applying a strategy based on metagenomic and metabolomic analyses, it was found that the enrichment of LPS biosynthesis suggests the potential role of GM in causing inflammation. To date, some findings raise the possibility that the low-grade inflammation and increase of gram-negative bacteria, especially *Prevotella* and *Klebsiella*, are likely responsible for hypertension pathology [43].

**Figure 1:**
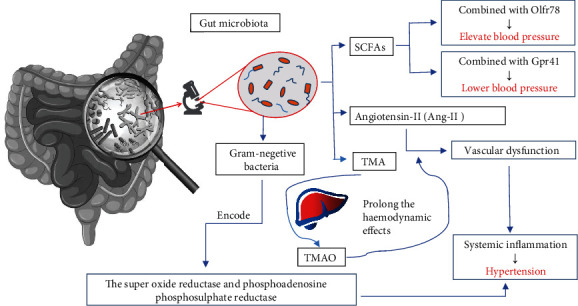
The effect of GM on blood pressure.

The specific mechanisms by which GM regulate blood pressure may be as follows. The first is that the SCFAs produced by the intestinal flora regulate blood pressure through a variety of pathways and receptors such as olfactory receptor 78 (Olfr78) and G protein-coupled receptor orphan type (Gpr41). SCFAs can increase the pressure when used in conjunction with the former and reduce the pressure when used in conjunction with the latter. Second, it has been established that intestinal flora facilitates vascular dysfunction induced by angiotensin-II (Ang-II), which further promotes systemic inflammation and consequently hypertension. Trimethylamine-N-oxide (TMAO), a main metabolites of GM, does not directly regulate blood pressure but prolongs the haemodynamic effects of Ang-II. Third, it is determined that inflammation triggered by LPS has been identified as a potential feature of the pathogenesis of gram-negative bacteria such as *Prevotella* and is likely responsible for HTN pathology [44]. In addition, the superoxide reductase and phosphoadenosine phosphosulphate reductase encoded by *Prevotella copri* may contribute to the development of inflammation [44] (Figure 1).

### 3.2. Gut Microbiota and AS

The causes of AS are multifaceted. There are now various hypotheses regarding its mechanism, including lipid infiltration, thrombosis, smooth muscle cell cloning, inflammation, and infection. Atherosclerotic heart disease is associated with a high rate of morbidity and mortality, as well as high healthcare costs, which are continuing to rise. A metagenome-wide association study analysis of the fecal flora of AS patients and healthy controls revealed that the abundance of *Enterobacteriaceae* and *Streptococcus* in AS feces was significantly increased, while *Bacteroides* and *Prevotella* is relatively reduced. To further investigate the role of GM in AS, it is found that the abundance of *Streptococcus* is positively correlated with blood pressure, and the abundance of *Enterobacteriaceae* is positively correlated with myocardial indicators in function prediction [45]. In previous studies, there are a wealth of information about relation of infection, immune system, and AS. For example, 9 patients with AS and 10 healthy controls were compared, and analyses have found that the intestinal flora of AS patients contained more *Collinsella*, while healthy controls contained more *Roseburia* and *Eubacterium* [46]. According to the mechanism of epidemiological associations, pathogens can infect macrophages and promote foam cell formation, leukocyte recruitment, smooth muscle proliferation, and disease progression, all of which are markers of AS [47]. These infections are supposed to result in the development of a AS by direct invasion of the microbiota. In addition to direct invasion, the microbiota response strongly from immune mediators which have been found to be involved in the development of AS. The specific mechanism is related to prominent inflammatory responses caused by recognition and engagement of toll-like receptors and nucleotide-binding oligomerization domain- (NOD-) like receptors and bacterial products such as lipopolysaccharides and peptidoglycans [48] [49].

There are three pathways by which GM might affect AS. First, local or distant infections might cause a harmful inflammatory response that aggravates plaque development or triggers plaque rupture [50]. Second, metabolism of cholesterol and lipids by GM can affect the development of atherosclerotic plaques [47]. Third, diet and specific components that are metabolized by GM can have various effects on AS [51]; for example, dietary fibre is beneficial for promoting the growth of beneficial commensal bacteria and thereby limiting the growth of opportunistic pathogen *Enterobacteriaceae* and increasing gut barrier-protecting *Bifidobacteriaceae*, with concomitant improving insulin sensitivity and lipid profile [52] [53], whereas the bacterial metabolite TMAO is considered harmful [54]. The related mechanism has been shown in previous studies. Metabolite TMAO promotes AS which is oxidized in the liver from trimethylamine (TMA) that is produced by the gut microbiota metabolizes choline, phosphatidylcholine, and L-carnitine [55] [56]. In addition, the studies on the changes of GM and the related mechanisms of AS suggest a potential therapeutic target for the prevention and treatment of AS; for instance, inhibition of gut microbiota-dependent TMAO production has been demonstrated to be a promising strategy for the treatment of AS.

GM regulates blood pressure by producing SCFs, Ang-II, and TMA which turn into TMAO in liver. And these three pathways are interrelated and interact with each other. It leads to hypertension when SCFAs combined with Olfr78 or there is systemic inflammation caused by vascular dysfunction. Meanwhile gram-negative bacteria and TMAO can promote the process.

### 3.3. GM and HF

HF is the final stage of many cardiovascular diseases. It is caused by various acute heart damages and subsequent abnormalities in compensatory mechanisms and pathogenic processes. The destruction of intestinal barrier function and the change of GM composition may lead to abnormal production and absorption of microbial metabolites in patients with HF. Imbalance of intestinal microbial metabolites and intestinal epithelial dysfunction may lead to cardiac dysfunction, inflammation, malnutrition, and other diseases in patients with HF [57]. HF leads to changes in blood flow such as insufficient tissue perfusion and gastrointestinal congestion, which can change body's intestinal morphology, permeability, and abundance and composition of GM, which in turn damages the intestinal barrier function, stimulates the inflammatory response, and accelerates its pathological development [58]. Takehiro et al. compared the GM of 12 patients with HF and 12 healthy controls based on 16S rRNA gene sequencing technology and found that *Eubacterium rectale* and *Dorea longicatena* were less abundant in the GM of HF patients than that in healthy control subjects [59]. And a decrease in the genus *Faecalibacterium* has been observed in HF patients. As for *Blautia*, *Faecalibacterium prausnitzii* was identified as an anti-inflammatory commensal, and its reduced abundance has been shown to be associated with impaired intestinal permeability [60] [61]. Takehiro et al. then confirmed the reduction of *Faecalibacterium prausnitzii* as an essential characteristic of the GM in HF patients [59]. Overall, these studies provide a strong support for the role of GM in HF patients.

**Figure 2:**
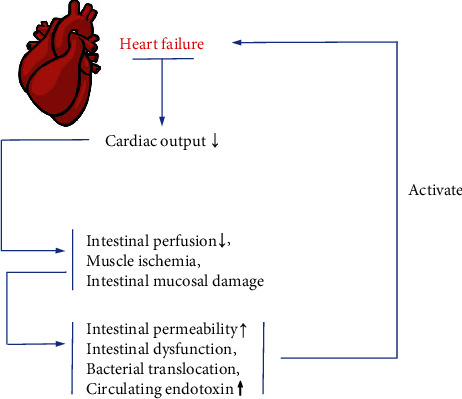
Mechanisms of HF.

The decrease of cardiac output in patients with HF can lead to decreased intestinal perfusion, muscle ischemia, and intestinal mucosal damage. And these changes will cause increased intestinal permeability, intestinal dysfunction, bacterial translocation, and increased circulating endotoxin, triggering inflammation related to HF and aggravating its pathogenesis [62] (Figure 2).

The accumulating evidence has demonstrated that imbalances in the composition and function of GM, referred to as dysbiosis, might change the gut immune system significantly. In addition, the direct regulation of GM is mainly through the immune system and hormone secretion. The metabolites of GM act on target organs, similar to endocrine organs of human hosts. However, the exact mechanism of these pathways remains to be specifically elucidated [63].

HF can lead to decreased intestinal perfusion, muscle ischemia, and intestinal mucosal damage because of the decreasing cardiac output. And these changes will cause increased intestinal permeability, intestinal dysfunction, bacterial translocation, and increased circulating endotoxin, thus activating inflammation related to HF and aggravating the pathogenesis of HF.

## 4. Effects of PA on CVD through Modulation of the Intestinal Microflora and Functions

During the past decades, there has been an increasing interest in studies for the effects of PA on the CVD. As mentioned above, diversity, distribution of the GM, and its functions on the metabolism and chronic systematic inflammation have been found to be impacted by physical exercise. Meanwhile, several experiments have demonstrated the relationship between CVD and the GM as well as its functions. Taken together with our previous studies, we can deduce a hypothesis that PA can influence CVD through alterations of the intestinal microflora and functions. Here, we will provide correlational research.

### 4.1. Impact of PA on CVD

Strong scientific evidence have supported that regular exercise is beneficial for the prevention and management of CVD and also has a positive intensity-related effect on various cardiovascular risk factors, such as hyperlipidemia, hypertension, abdominal obesity, diabetes, and psychosocial factors. Individuals with a high PAR such as forager-horticulturalist, pastoralist, and traditional farming populations have a low incidence of coronary atherosclerosis and almost no risk factors for CVD [64]. Firstly, exercise is associated with increased flow-mediated shear stress on the artery walls. Secondly, serum C-reactive protein levels are decreasing during exercise. Furthermore, endurance exercise decreases blood pressure and serum triglyceride (TG) levels and improves high-density lipoprotein cholesterol levels (HDL-C), insulin sensitivity, and glucose homeostasis. In addition, protective effects against CVD is positively related to exercise intensity [65]. However, some researchers are currently questioning the benefits of very high doses of regular physical activity or exercise.

Nonetheless, technological improvements over the past decades have led to a trend toward inactivity lifestyle, resulting in a suboptimal cardiovascular phenotype and an increasing risk of cardiometabolic disorders. As a result, PA must be increased to prevent cardiovascular disease.

### 4.2. Relationship between GM, PA, and CVD

Till now, the biological mechanisms underpinning the cardiovascular benefits of an active lifestyle are still not well understood. Speculated from previous studies, we can summarize that there are some relations between GM, physical exercise, and CVD.

Discoveries in the past decade have shed light on an idea that an unhealthy phenotype of GM might increase the risk of CVD via several mechanisms, such as increased production of TMAO [66], increased endotoxaemia [67], increased bacterial translocation to carotid arterial plaques [68], and increased blood pressure [69]. For example, the atherosclerotic plaque areas in female rats have a positive correlation with *Clostridiales*, *Ruminococcus*, and *Lachnospiraceae* and have a negative correlation with the *S24–7* family of *Bacterioidetes* [70]. Also, SCFAs have specific functions of affecting epithelial cell transport and metabolism, growth, and differentiation and controlling lipid and carbohydrate metabolite in hepatocytes and providing energy sources, which are all related to CVD [66].

Physical exercise, through improving the diversity and the construction of the GM, altering the abundance of certain bacterial species like *Faecalibacterium prausnitzii* and changing the intestinal metabolites, can modulate GM towards a healthy phenotype, further influencing the CVD. For instance, after endurance running, the diadenosine polyphosphates (APnAs, *n* = 3–6) released into the circulation were characterized to function as vasoconstrictors and to be involved in cardiovascular physiology [16]. Moreover, many studies have showed that physical activity is anti-inflammatory and protective against developing chronic inflammatory diseases which are cardiovascular risk factors, via GM and intestinal permeability [66].

Overall, the relation between the GM, PA, and CVD is still indistinct, and it needs to be investigated intensively, as well as the biological mechanisms.

## 5. Conclusions

Although there is still a wealth of mechanisms and connections remained to be studied, the current studies demonstrate that it is time to consider PA as therapeutic strategy for the management of CVDs for that PA affects CVD through regulation of the GM and intestinal function.

In summary, PA can modulate the GM towards a healthy phenotype by increasing the diversity and the construction of the GM, affecting the abundance of certain bacterial species and changing the intestinal metabolites, to influence the CVD.

For hypertension, the intestinal flora produces the SCFAs and TMAO which facilitates the function of Ang-II and triggers inflammation to increase or decrease blood pressure. For AS, metabolism of cholesterol and lipids by GM can affect the development of AS plaques. Furthermore, infections might cause a harmful inflammatory response that aggravates plaque development or triggers plaque rupture. For HF, intestinal dysfunction, bacterial translocation, and increased circulating endotoxin will activate inflammation related to HF and aggravate the pathogenesis of HF.

All up, we believe that an article summarizing the relevant relationship between PA and GM might be of assistance for clinical medicine. As we gain a further understanding of regulation of the GM and intestinal function, researchers and scholars will one day target therapies for CVD patients towards GM and PA successfully and further improve the clinical care of patients with CVD.

## Data Availability

Data are available.
